# Barriers, facilitators, and implementation strategies for pharmacogenomics in community pharmacies: a cross-sectional survey among local champions in pharmacies and key opinion leaders in pharmacogenomics

**DOI:** 10.1007/s11096-025-02022-x

**Published:** 2025-10-23

**Authors:** Pantea Kiani, Pierre M. Bet, Naomi T. Jessurun, Petra Hoogland, Jeroen Mentink, Jesse J. Swen, Sander D. Borgsteede

**Affiliations:** 1 Division of Pharmacology, Utrecht Institute for Pharmaceutical Sciences, Utrecht University, 3584CG Utrecht, The Netherlands; 2 Department of Clinical Pharmacology and Pharmacy, Amsterdam University Medical Center, Amsterdam, The Netherlands; 3 Personalized Medicine Program, Amsterdam Public Health Research Institute, Amsterdam, The Netherlands; 4 Netherlands Pharmacovigilance Centre Lareb, ‘s-Hertogenbosch, The Netherlands; 5 Department of Pharmaceutical Affairs, Service Apotheek Beheer B.V., Enter, The Netherlands; 6 Community Pharmacist, Borrendamme Pharmacy, Zierikzee, the Netherlands; 7 Department of Clinical Pharmacy and Toxicology, Leiden University Medical Center, Leiden, The Netherlands; 8 Department of Clinical Decision Support, Health Base Foundation, Houten, The Netherlands; 9 Unit PharmacoTherapy, Epidemiology and Economics, Groningen Research Institute of Pharmacy, University of Groningen, Groningen, The Netherlands

**Keywords:** CFIR, Community pharmacy, Implementation barriers, Implementation strategies, Pharmacogenomics

## Abstract

**Introduction:**

Pharmacogenomics (PGx) tailors drug treatments to an individual’s genetic profile and contributes to improved efficacy and reduced adverse drug reactions. Community pharmacists have shown interest in PGx, and Dutch pharmacists have been early adopters in applying PGx guidelines, particularly through integration of the Dutch Pharmacogenetics Working Group recommendations. Despite growing evidence of its benefits, large-scale implementation in community pharmacies remains limited. This raises an important question for global stakeholders: if PGx adoption is constrained even in a system with robust infrastructure and guidelines, what lessons can be drawn for broader implementation?

**Aim:**

To examine current PGx practices in leading Dutch community pharmacies and to identify key barriers, facilitators, and implementation strategies for integrating PGx into routine pharmacy care.

**Method:**

A cross-sectional survey was conducted among Dutch pharmacy professionals with experience in PGx implementation. Participants were categorized as key opinion leaders (KOLs), involved in national PGx policymaking, research, or representing academia or professional organizations, or local champions (LCs), defined as practicing pharmacists directly involved in local PGx implementation in hospital or community pharmacies. The questionnaire was retrospectively mapped to Consolidated Framework for Implementation Research domains. Quantitative data were analyzed descriptively and qualitative responses were inductively thematically grouped to contextualize findings.

**Results:**

Of the 67 invited professionals, 46 completed the questionnaire (response rate: 69%). Among respondents, 70% were LCs and 30% KOLs. Among KOLs (n = 14), the most frequently cited barriers included costs (79%, n = 11/14), inadequate Information and Communication Technology (ICT) support (29%, n = 4/14), and legal or regulatory uncertainty (29%, n = 4/14). A positive view on the clinical value of PGx was reported by 81% of LCs (n = 26/32) and 93% of KOLs (n = 13/14). Suggested facilitators by LCs included improved ICT infrastructure (56%, n = 18/32), enhanced education and training (44%, n = 14/32), and stronger interdisciplinary collaboration (25%, n = 8/32).

**Conclusion:**

PGx implementation in Dutch pharmacies is hindered by structural barriers such as fragmented ICT and lack of reimbursement, despite strong professional support. Embedding PGx into pharmacy workflows and aligning policy, infrastructure, and education are essential. These findings may inform broader efforts to integrate PGx into pharmacies across diverse systems.

**Supplementary Information:**

The online version contains supplementary material available at 10.1007/s11096-025-02022-x.

## Impact statements

- This study identifies practical barriers and facilitators to implementing PGx in community pharmacies, including ICT infrastructure, reimbursement, and training. The findings can support the development of targeted implementation strategies to enable effective and sustainable integration of PGx into routine pharmacy practice.

## Introduction

Pharmacogenomics (PGx), the study of how genetic variation influences an individual’s response to medications, is increasingly recognized as a key pillar of precision medicine. By individualizing pharmacotherapy based on genetic profiles, PGx holds the potential to improve drug efficacy, reduce adverse drug reactions, and optimize treatment outcomes [1–5]. Pharmacogenetics, traditionally focused on single gene–drug interactions, is often used interchangeably with PGx; throughout this manuscript, the term PGx is used consistently.

International studies have demonstrated the feasibility and clinical utility of PGx implementation in primary care and community pharmacy settings, particularly in managing chronic diseases and psychiatric conditions [6–9]. In regions such as North Cyprus, Yemen and Austria, pharmacists generally hold positive attitudes toward PGx, yet they report barriers including insufficient training, lack of reimbursement, and limited digital support systems [10–12]. Similar patterns are seen in Australia, Canada and North America [13–15]. Pharmacist-led PGx initiatives in these regions have been associated with improved patient outcomes and satisfaction; however, concerns persist regarding infrastructure, cost‐effectiveness, and integration into existing workflows [10–14].

Pharmacists are widely considered well-positioned to support PGx implementation due to their accessibility and pharmacotherapeutic expertise [16–20]. In the Netherlands, community pharmacies, both independent and chain-affiliated, operate under a nationally regulated system that grants pharmacists considerable autonomy. In this context, community pharmacists play a key role in primary care drug treatment. Beyond the distribution of medications, their responsibilities include ensuring the safety of prescribed drugs, optimizing pharmacotherapy in collaboration with general practitioners and medical specialists, and advising patients on medication usage. These activities have been shown to enhance patient safety and reduce hospitalizations related to medication problems. Patients typically remain loyal to a single pharmacy, supporting continuity of pharmaceutical care and effective medication surveillance [21]. National guidelines issued by the Dutch Pharmacogenetics Working Group (DPWG) are integrated into nearly all pharmacy information systems, enabling real-time clinical decision support. Early adopters have expressed willingness to use PGx in clinical decision-making, making the Netherlands one of the most advanced countries in Europe in terms of infrastructure and guideline availability [8].

Despite this favorable context, PGx has yet to become embedded in routine Dutch pharmacy workflows. This paradox underscores a broader issue: if implementation proves challenging even in a highly structured and supportive system, what barriers might be anticipated elsewhere and what insights can be gained for countries still at earlier stages of adoption? Structural barriers commonly reported in systematic evaluations and literature [22–29]—such as lack of reimbursement, unclear procedures, digital fragmentation, and limited interprofessional collaboration—have not been systematically explored in the Dutch community pharmacy setting. Moreover, little is known about how these obstacles are experienced by those most closely involved in implementation.

As such, the Netherlands offers a valuable case study for other healthcare systems: by examining a high-resource context that has not yet achieved full PGx integration, this study provides transferable insights into the structural conditions and policy mechanisms needed for successful adoption.

With accumulating evidence for PGx utility [30–34] and the momentum from EU initiatives such as the U-PGx PREPARE study [30], understanding the practical drivers of PGx adoption is increasingly urgent. Lessons learned from the Dutch context may inform international efforts to embed PGx into pharmacies, particularly in countries facing comparable infrastructural, regulatory, or organizational challenges.

### Aim

The aim of this study was to examine current PGx practices in leading Dutch community pharmacies and to identify key barriers, facilitators, and implementation strategies for integrating PGx into routine pharmacy care, structured according to the Consolidated Framework for Implementation Research (CFIR) framework.

## Method

### Study design

This study used a cross-sectional survey. The survey was conducted using a structured questionnaire in January 2024 among the KOLs and LCs identified by the working group. We described the manuscript according to the guidelines for strengthening the reporting of observational studies in epidemiology (STROBE) [35], with the checklist for observational studies as Supplementary material Appendix I.

### Participants

PGx-experienced professionals were purposively selected by the author team, all members of a national PGx working group. Two subgroups were defined: key opinion leaders (KOLs), involved in national PGx policymaking, research, or representing academia or professional organizations, and local champions (LCs), defined as practicing pharmacists directly involved in local PGx implementation in hospital or community pharmacies. All participants had verifiable experience in PGx and included clinical and hospital pharmacists, academic professionals, and representatives of professional organizations. In addition to community pharmacists, we purposively invited hospital pharmacists who were demonstrably involved in cross-sector PGx implementation (e.g., co-developing PGx workflows with community pharmacies, contributing to shared ICT/decision-support infrastructures, and co-authoring or disseminating national guidelines).

Participants were identified through professional networks of the working group. To minimize selection bias, a snowball sampling approach was employed, enabling invited individuals to nominate additional eligible colleagues. No financial incentives were provided.

Recruitment was conducted via personalized email invitations containing a brief study description, an informed consent statement, and a link to the online survey platform. No formal sample size was predefined. Recruitment continued until a sufficiently diverse and information-rich sample was achieved, capturing perspectives across clinical and strategic domains of PGx implementation.

### Questionnaire development

The questionnaire was developed by two members of the working group (PK, SB), based on key topics identified by the full team. The remaining members reviewed the instrument and participated in pilot testing. This included professionals working in community pharmacy settings (JM, PH), hospital pharmacy (PB and JS), and pharmacy and society not related to patient care (NJ). Suggestions for removing questions, adjusting answer categories, and changing the order and wording of questions were implemented. A translated version of the questionnaire is provided in Appendix II.

The questionnaire was structured into several sections. The first section gathered demographic and professional background information. Based on participants’ responses, a routing mechanism directed them to role-specific modules: LCs answered questions related to clinical PGx application, while KOLs received items concerning policy, infrastructure, and system-level implementation. The structured questionnaire included both closed- and open-ended items. Participants were automatically routed to KOL- or LC-specific sections based on their role.

Subsequent sections assessed perceived barriers, facilitators, roles, collaboration, and implementation experiences regarding pharmacogenetics. The questionnaire included a variety of item formats: multiple-choice questions, frequency-based response scales (e.g., “Never” to “Always”), 3-point Likert-type scales (e.g., “Inadequate” to “Adequate”, “Negative” to “Positive”), and dichotomous questions (“Yes”/“No”). Many items were supplemented with optional open-text fields to allow respondents to elaborate, providing additional qualitative insights that enriched the quantitative data.

### Data collection and analysis

The questionnaire was administered online via SurveyMonkey (www.surveymonkey.com) and remained open between 9 and 21 February 2024. Responses were exported to Microsoft Excel for Mac (version 16.97.2, Build 25052611) for descriptive analysis. For all closed-ended items, the number of respondents, percentages and missing values were reported in tables. Optional open-text fields were analyzed qualitatively to capture additional insights.

Open comments were analyzed for each question using an inductive approach meant. All comments were listed, and frequently mentioned or representative comments were included in the tables as topics. No qualitative analysis software or predefined coding framework was used; one of the researchers (SB) conducted the analysis and proposed the selected comments, another researcher (PK) reviewed and agreed on the final selection. Remarks and suggestions related to the main topics—barriers, facilitators, and strategies—were analyzed deductively according to the corresponding questions. All responses were listed by one of the authors (SB), and within each main topics, the responses were categorized inductively into themes that were mentioned more than 5 times as barrier, facilitator or strategy. The categorization into main topics and inductive coding was checked by another member of the Working Group (PK) [36]. Remarks and suggestions that were exemplary for a specific topic were added to the quantitative findings for illustration.

Although the questionnaire was not originally developed using CFIR, all items were retrospectively mapped to CFIR’s five domains—Innovation (Intervention Characteristics), Inner Setting, Outer Setting, Characteristics of Individuals, and Implementation Process—to facilitate structured analysis of PGx-related barriers and facilitators [37]. By mapping each question to a specific CFIR domain, the study ensured a structured analysis of barriers and facilitators to pharmacogenomics implementation (see for mapping Appendix III).

To maintain respondent perspectives while enhancing readability, result tables include synthesized keywords or thematic labels rather than verbatim quotations. The survey was conducted in Dutch, and participation was voluntary.

### Ethics approval

Ethics approval was not required under the Dutch Medical Research Involving Human Subjects Act (WMO), as this study only involved healthcare professionals, and did not affect personal patient data [46]. In January 2024, data were collected anonymously and stored in compliance with relevant privacy regulations.

## Results

### Study population

Of the 67 healthcare professionals invited, 46 completed the questionnaire (response rate: 69%, n = 46/67). As shown in Table 1, the sample consisted of both LCs (70%, n = 32/46) and KOLs (30%, n = 14/46), with diverse backgrounds in community and hospital pharmacies, education, technology, and professional organizations. Most respondents had substantial professional experience, and the majority were female.

**Table 1:** Study population (professional background and experience) (n = 46)

Category	Subcategory	Number of respondents N = 46
Professional background	Local champions—community pharmacist	25 (54%)
	Local champions—hospital pharmacist	7 (15%)
	Key opinion leaders	14 (30%)
	Missing	0 (0%)
Experience	0–5 years	10 (22%)
	6–15 years	11 (24%)
	> 15 years	23 (50%)
	Missing	2 (4%)
Gender	Female	26 (57%)
	Male	19 (41%)
	Other	1 (2%)
	Missing	0 (0%)

Below, the main findings of the questionnaire are presented according to the CFIR-domains Innovation (intervention characteristics), outer setting, inner setting, characteristics of the individuals, and implementation process [37].

### Innovation

Most LCs (81%, n = 26/32) were confident in PGx’s healthcare benefits. The KOLs identified key barriers, including costs (79%, n = 11/14), legal obstacles (29%, n = 4/14), and a lack of adequate ICT support (29%, n = 4/14) (Table 2). LCs suggested several ways to address these barriers, focusing on improving technology systems (56%, n = 18/32) and providing more training and education (44%, n = 14/32).

**Table 2:** Questions related to CFIR domain intervention characteristics,

Question	Local champions (n = 32)	Key opinion leaders (n = 14)	Comments and remarks in questionnaire
What is your vision on the position of PGx in healthcare?			
Positive	81% (n = 26)	93% (n = 13)	Improved quality, reduced costs, and quicker results anticipated
Negative	3% (n = 1)	7% (n = 1)	
Missing	16% (n = 5)	0% (n = 0)	
How do you see the future of PGx?			
Positive	81% (n = 26)	Not applicable^a^	Enhanced role of pharmacists, potential for cost reduction, and better patient outcomes
Uncertain	3% (n = 1)		
Missing	16% (n = 5)		
What obstacles do you see for the implementation of PGx?			
Costs	Not applicable^a^	79% (n = 11)	Barriers include funding issues, lack of ICT^b^ support, and insufficient knowledge
Legal issues		29% (n = 4)	
Lack of evidence		21% (n = 3)	
Other (a.o.lack of ICT^**b**^			
Support, n = 4/29%)		64% (n = 9)	
Missing		0% (n = 0)	
What initiatives or improvements will have the highest impact?			
Technological improvements	56% (n = 18)	Not applicable^a^	Emphasis on improving technology, education, and regional cooperation
Knowledge and education	44% (n = 14)		
Improved cooperation	25% (n = 8)		
Public information	22% (n = 7)		
Missing	16% (n = 5)		

^a^Local champions and key opinion leaders received a different set of questions

^b^Information and communication technology

### Outer setting (external factors)

LCs showed varying levels of awareness about PGx regulations. Among those who responded to this item (n = 27/32), 47% (n = 15/32) reported being familiar with the existing recommendations, 28% (n = 9/32) were somewhat familiar, and 9% (n = 3/32) were not familiar at all. Regarding financial barriers, only 13% (n = 4/32) of LCs who answered the question (n = 28/32) reported receiving any reimbursement for PGx-related services (Table 3). When asked about the adequacy of current regulations, 44% (n = 14/32) found them moderately sufficient, while 15% (n = 5/32) viewed them as inadequate. KOLs noted that non-professionals could play a significant role in raising public awareness about PGx, with 86% (n = 12/14) highlighting this potential.

**Table 3:** Questions related to CFIR domains: outer setting (external factors) and inner setting (organizational context)

Question	Local champions (n = 32)	Key opinion leaders (n = 14)	Comments and remarks in questionnaire
Outer setting			
Are you aware of current PGx regulations in the pharmaceutical sector?			
Yes	Not applicable^a^	43% (n = 6)	Need consistent regulatory frameworks
Partially		21% (n = 3)	
No		21% (n = 3)	
Missing		14% (n = 2)	
Are you aware of current guidelines and protocols on PGx?			
Yes	47% (n = 15)	Not applicable^a^	Need for clearer guidelines
Partially	28% (n = 9)		
No	9% (n = 3)		
Missing	16% (n = 5)		
How adequate are current regulations for PGx, in your opinion?			
Adequate	19% (n = 6)	Not applicable^a^	Revision of existing regulations needed
Moderately adequate	44% (n = 14)		
Inadequate	16% (n = 5)		
Missing	19% (n = 6)		
Do you receive reimbursement for PGx-related services?			
Yes	13% (n = 4)	Not applicable^a^	New reimbursement models suggested that overcoming financial barriers is critical
No	72% (n = 23)		
Missing	15% (n = 5)		
What is the importance of non-professionals in promoting PGx?			
Important	Not applicable^a^	86% (n = 12)	Potential role in increasing public awareness and involvement
Not important		7% (n = 1)	
Missing		7% (n = 1)	
Inner setting			
Do you store complete PGx-reports?			
Yes	75% (n = 24)	Not applicable^a^	Need for consistent data storage
No	9% (n = 3)		
Missing	16% (n = 5)		
How is PGx information recorded?			
As contraindication	59% (n = 19)	Not applicable^a^	Integration into existing systems recommended
As lab result	9% (n = 3)		
Both	9% (n = 3)		
Missing	23% (n = 7)		
What ICT^b^ problems do you experience concerning the implementation of PGx?			
Manual labor	53% (n = 17)	Not applicable^a^	High manual workload, difficulties in data exchange
Difficult to identify at-risk patients	16% (n = 5)		
Exchange digital information	50% (n = 16)		
Missing	16% (n = 5)		
How is PGx information communicated with other ICT^b^ systems?			
On paper	56% (n = 18)	Not applicable^a^	Electronic communication between systems needed
By email	53% (n = 17)		
Automatically as contraindication	34% (n = 11)		
Missing	12.5% (n = 4)		

^a^Local champions and key opinion leaders received a different set of questions

^b^Information and communication technology

### Inner setting (organizational context)

The majority of LCs (75%, n = 24/32), indicated they store complete PGx reports but encounter notable barriers with ICT systems. Information is often recorded as contraindications (59%, n = 19/32), and half of the respondents (50%, n = 16/32) reported difficulties in sharing digital information. Communication with other healthcare providers primarily relies on paper-based methods (56%, n = 18/32) and email (53%, n = 17/32) (Table 3). Respondents most frequently mentioned manual processes (53%, n = 17/32) and difficulty identifying at-risk patients (16%, n = 5/32) as barriers. In the open comments the respondents mentioned how improved ICT systems could support their efforts in implementation of PGx: improved exchange of PGx test results by data-sharing between laboratories and with primary and secondary care providers; and automatic translation of these PGx test results into relevant alerts for clinical decision support systems.

### Characteristics of the individuals

LCs were the main providers of PGx advice, with 44% (n = 14/32) being solely responsible and 41% (n = 13/32) sharing this responsibility with pharmacy technicians. 72% (n = 23/32) of LCs reported advising fewer than five patients per week (Table 4). The most frequently targeted patient groups by the LCs were those with cardiovascular conditions (41%, n = 13/32) and psychiatric disorders (44%, n = 14/32).

**Table 4:** Questions related to CFIR characteristics of the individuals

Question	Local champions (n = 32)	Comments and remarks in questionnaire
How many patients do you advise on PGx each week?		
< 1	34% (n = 11)	Encourage tracking and increasing patient advice based on PGx
1–5	31% (n = 10)	
> 5	16% (n = 5)	
Missing	13% (n = 4)	
How do you inform your patients about PGx?		
Personal at the counter	59% (n = 19)	Enhance patient education efforts, especially in personalized settings
Written	25% (n = 8)	
By phone	31% (n = 10)	
Website	22% (n = 7)	
App	9% (n = 3)	
Missing	16% (n = 5)	
Who is responsible for advice on PGx?		
Pharmacist	41% (n = 13)	Establish clear roles within pharmacies
Pharmacy technician	3% (n = 1)	
Both	41% (n = 13)	
Missing	13% (n = 4)	
Do you approach patients actively regarding PGx?		
Yes	38% (n = 12)	Strategies to actively engage patients
No	47% (n = 15)	
Missing	16% (n = 5)	
Do you advise on medication safety in patients with a PGx profile?		
Yes	78% (n = 25)	Practical tools for integrating PGx into medication reviews are needed
No	3% (n = 1)	
Other	3% (n = 1)	
Missing	16% (n = 5)	
Do you aim your activities on a specific patient population?		
Cardiovascular	41% (n = 13)	Patient-specific strategies for PGx use
Psychiatric	44% (n = 14)	
Pain	22% (n = 7)	
Oncology	13% (n = 4)	
Other	38% (n = 12)	
Missing	19% (n = 6)	

### Process

Most LCs (66%, n = 21/32) reported handling communication about PGx with other healthcare professionals on a case-by-case basis, while only 19% (n = 6/32) relied on formal local agreements. When communicating with patients about PGx, in-person discussions at the pharmacy counter were the most common method (59%, n = 19/32), followed by phone consultations (31%, n = 10/32) and written materials (25%, n = 8/32) (Table 5). 34% (n = 11/32) of LCs indicated that patients were generally unaware of PGx. With respect to collaboration between healthcare providers, respondents suggested in their comments that non-healthcare professionals could play a role in raising public awareness about PGx.

**Table 5:** Questions related to CFIR domain process

Question	Local champions (n = 32)	Key opinion leaders (n = 14)	Comments and remarks in questionnaire
How do you assess your PGx expertise level?			
Beginner	3% (n = 1)	Not applicable^a^	Comprehensive training programs required
Intermediate	63% (n = 20)		
Expert	19% (n = 6)		
Missing	15% (n = 5)		
How do you communicate with professional colleagues about PGx?			
By local agreements	19% (n = 6)	Not applicable^a^	Encouragement to establish local/regional hubs for better coordination
Case by case	66% (n = 21)		
Other	3% (n = 1)		
Missing	15% (n = 5)		
Are patients well-informed about PGx?			
Yes	3% (n = 1)	Not applicable^a^	Increase efforts to inform patients
Sometimes	47% (n = 15)		
No	34% (n = 11)		
Missing	15% (n = 5)		
Is there sufficient awareness concerning PGx among non-healthcare professionals?			
Yes	Not applicable^a^	0.0	Awareness campaigns needed
Partial		43% (n = 6)	
No		50% (n = 7)	
Missing		7% (n = 1)	
How do you rate current communication between healthcare professionals and non-professionals to PGx?			
Effective	Not applicable^a^	6% (n = 2)	Standardized communication protocols needed
Neutral		16% (n = 5)	
Not effective		19% (n = 6)	
Missing		7% (n = 1)	

^a^Local champions and key opinion leaders received a different set of questions

### Analysis of open-ended questions concerning barriers, facilitators and strategies

Topics that were illustrative of the quantitative findings have been added to Tables 2, 3, 4 and 5. Typical examples from the quotes provided in the open questions are included in Appendix IV. The open answers for the barriers and facilitators experienced and the strategies for implementation that were suggested are shown in Table 6. Barriers consisted of reimbursement challenges and lack of standardization, while visibility, patient engagement and multidisciplinary cooperation were experienced as facilitator. Respondents mentioned ICT both as a barrier (e.g., lack of interoperability) and as a facilitator (e.g., potential for integration with clinical decision support systems). Suggested strategies for implementation were the development of educational programs and regional network, integration in guidelines, and the availability of toolkits and best practices.

**Table 6:** Comprehensive overview of remarks and suggestions of respondents

Barriers	
Reimbursement challenges	
Data sharing issues	
Inconsistent PGx data access	
Lack of standardized reporting	
Facilitators	
Increased visibility	
Digital tools	
Patient engagement	
Multidisciplinary cooperation	
Strategies	
Education programs	
Master-level training	
Integration in clinical guidelines	
Toolkits and best practices	
Regional networks	

In terms of interdisciplinary collaboration, participants noted that non-healthcare professionals—such as those involved in communication, education, or technology—could play a supportive role in raising public awareness about the potential benefits of PGx.

Regarding education and implementation in daily practice, respondents expressed a need for more practical and context-specific tools, such as workflows, templates, or checklists, to facilitate the routine application of PGx in pharmacy settings.

## Discussion

### Statement of key findings

This study examined the barriers, facilitators, and strategies for implementing pharmacogenomics (PGx) in community pharmacies in the Netherlands. Responses from 46 healthcare professionals, including 32 LCs, were retrospectively categorized using the CFIR [37] to structure and interpret the findings.

Our key findings identified financial constraints, inadequate ICT infrastructure, and insufficient knowledge as the main barriers to PGx implementation. Notably, only 13% (n = 4/32) of LCs indicated that current reimbursement mechanisms were adequate. In addition, just 16% (n = 5/32) reported advising more than five patients per week on PGx, suggesting that routine PGx-application in daily practice remains limited despite its perceived clinical utility. “Inadequate ICT infrastructure” primarily refers to the lack of interoperability between primary and secondary care IT infrastructures, which prevents seamless exchange of PGx results. In addition, laboratories currently do not have the capability to electronically transmit PGx results directly into pharmacy information systems. Facilitators included professional training, clearer policies, and improved infrastructure, with 44% (n = 14/32) of LCs suggesting enhanced education and 56% (n = 18/32) emphasizing better ICT systems as solutions.

### Interpretation

Although this study was conducted in the Netherlands, the findings have broader international relevance. Many of the identified barriers—including fragmented ICT systems, insufficient reimbursement models, and lack of pharmacist-specific training—are reported across healthcare systems in countries such as the United Kingdom, Ireland, Australia, Canada, and the United States [13–16].

By retrospectively applying CFIR domains, this study illustrates how structured frameworks can support systematic identification of barriers and facilitators in PGx adoption. Among the most prominent challenges were financial constraints. While PGx testing itself is relatively low-cost, particularly compared to other diagnostics, pharmacist services, such as medication review, data interpretation, and counselling are underfunded [1, 4, 5]. These services represent a substantial workload and are not adequately reimbursed. On a population level, costs may increase with broader testing, yet studies have shown cost-effectiveness through reduced adverse drug reactions and hospitalizations [2, 33, 34].

Technical barriers were also widely reported. Fragmented ICT systems and the lack of decision-support tools hinder efficient data processing and integration. This aligns with international literature pointing to digital fragmentation as a critical bottleneck [3, 38]. The development of interoperable systems and embedded clinical decision support could reduce administrative burden and support uptake [5, 33].

Knowledge gaps were evident as well. For example, fewer than half of the LCs (47%, n = 15/32) were familiar with relevant PGx regulations, indicating a need for targeted training. These results are consistent with prior studies highlighting similar educational deficiencies [33, 34, 39, 40]. Comparable findings have emerged in studies from Australia and Canada, where pharmacists have expressed interest in PGx but report feeling underprepared for clinical implementation due to limited exposure and lack of structured education programs [14, 15].

Despite these barriers, the Dutch context provides several structural advantages. The integration of PGx into national guidelines (via DPWG), the pharmacist's role in medication surveillance, and the use of decision-support systems have supported early adoption. Nevertheless, implementation remains limited, suggesting misalignment between policy, reimbursement, and infrastructure. This paradox, where high structural readiness does not automatically lead to widespread clinical adoption, offers valuable insights for other countries. Even in a well-organized healthcare system with technical capacity and professional willingness, PGx integration may stall without aligned financial and regulatory frameworks.

In countries lacking such national coordination—such as parts of Eastern Europe or decentralized systems like the US—the challenges are likely to be even greater. In the US, for instance, PGx services are often fragmented, and reimbursement varies widely across states and insurers [13]. This underscores the limitations of bottom-up efforts. While early adopters and professional initiatives are valuable, systemic obstacles such as unclear reimbursement structures or regulatory gaps require top-down solutions. Prior studies have shown that without national mandates, financial incentives, and integrated workflows, PGx implementation often remains restricted to pilot projects [20, 23, 26, 28]. The Dutch case can therefore serve as both a blueprint and a warning: robust infrastructure alone is insufficient to secure routine adoption. A combined approach, top-down policy alignment and bottom-up professional engagement, is needed.

Even in a well-resourced and supportive environment, implementation barriers persist. The Dutch experience illustrates that both professional motivation and policy alignment are essential for sustainable adoption. This dual requirement echoes findings from implementation studies in countries such as Australia and Canada, where lack of national leadership has similarly slowed integration despite pilot success [14, 15]. This dual approach—combining grassroots engagement with structural support—may be key to achieving broader integration of PGx into community pharmacy. The insights from this study may therefore inform international implementation strategies.

In addition to the structural requirements of financial and policy alignment, our respondents emphasized several specific implementation strategies. First, the development of targeted educational programs and master-level training were seen as essential to address knowledge gaps and build pharmacists’ confidence in routine PGx use. Second, regional networks and collaborative hubs were suggested as a means to foster interdisciplinary exchange and create critical mass for implementation at the local level. Third, integration of PGx into existing national guidelines and protocols was considered crucial for standardization and legitimacy of practice. Finally, respondents highlighted the need for practical toolkits and best practices (e.g., standardized workflows, checklists, and communication templates) to facilitate translation of PGx into daily pharmacy routines. Taken together, these strategies provide actionable levers that complement broader policy alignment and professional motivation, and they may serve as directly transferable approaches for other healthcare systems aiming to embed PGx in community pharmacy.

### Strengths and weaknesses

A strength of this study is the inclusion of both KOLs, and LCs actively involved in PGx implementation, yielding a high response rate (69%, n = 46). The study captured diverse perspectives from both practicing pharmacists and professionals in social or administrative roles and was structured using the CFIR framework [37]. A further strength is that the study yielded not only perceived barriers and facilitators but also specific, practice-oriented implementation strategies, such as education, regional networks, guideline integration, and toolkits.

Nonetheless, the survey relied on a relatively small, selected and specialized sample, which limits generalizability to the broader community pharmacy population. However, this purposive sampling strategy was intentional, aiming to capture insights from individuals with substantial experience in PGx implementation in the Netherlands. As such, the findings should be viewed as indicative of leading practice contexts, rather than representative of all pharmacists. This focus on frontrunners also provides a ‘best-case scenario’ benchmark for other countries. Lessons from these early adopters can help identify which barriers remain even under favorable conditions.

To ensure diversity within this targeted group, participants were drawn from a range of roles and settings. The questionnaire was developed based on relevant literature and expert opinion but has not undergone formal validation procedures such as assessments of construct validity, criterion validity, or test–retest reliability. This is a common limitation in emerging research fields where validated instruments are lacking. While such questionnaires can provide valuable exploratory insights, the absence of validation may affect the interpretability and generalizability of findings, potentially introducing bias [41]. The qualitative comments and remarks presented in this study should be interpreted as illustrations of the quantitative findings and not as an equivalent to a qualitative study that would explore these concepts and underlying characteristics more rigorously.

Furthermore, due to the role-specific routing of the questionnaire, key topics—such as perceived barriers and implementation strategies—were addressed to only one group (either LCs or KOLs). While this enhanced relevance, it limited the ability to perform cross-group comparisons for these domains. Potential response bias was addressed through anonymized data collection and the inclusion of open-ended questions to encourage honest and nuanced feedback. Social desirability bias was further mitigated through the use of neutrally worded items and confidential participation.

The Dutch healthcare context—with its relatively advanced PGx infrastructure and supportive policy environment—may differ from less developed systems, which could limit the transferability of findings. In addition, the reliance on self-reported data introduces the possibility of recall bias or overestimation of knowledge and implementation success. Finally, the dual role of the PGx expert working group in both study design and participant selection may have introduced confirmation bias, although this was mitigated through the use of CFIR [37] and the application of snowball sampling to broaden participant inclusion. Nevertheless, by making visible where and why PGx implementation lags even in favorable conditions, this study contributes to broader understanding of what it takes to move beyond pilot phases in community pharmacy.

Our findings are rooted in the Dutch healthcare system which raises the question of whether similar PGx implementation is feasible in other healthcare systems. Even in a context with national guidelines, pharmacist engagement, and ICT infrastructures, barriers such as insufficient reimbursement and fragmented data exchange persist. This suggests that also other systems, certain prerequisites are indispensable: sustainable funding models that include pharmacist services, basic ICT interoperability for secure data exchange, targeted professional training, and integration of PGx into clinical guidelines. While these elements do not all need to be in place from the outset, incremental progress in these areas is essential to move beyond pilot projects. The Dutch case therefore illustrates both the opportunities and the risks: professional motivation is important, but without systemic alignment, large-scale implementation is unlikely to succeed.

### Further research

Advancing reimbursement structures will require coordinated efforts between pharmacists and policymakers to establish sustainable funding models that cover both PGx testing and the associated pharmaceutical services, such as counselling, clinical interpretation, and medication reviews [39, 40, 42].

Larger, well-powered studies are needed to assess the generalizability of current findings across different pharmacy settings and healthcare systems. To address persistent technical barriers, future research should explore the development and implementation of interoperable ICT platforms and clinical decision-support tools that can seamlessly integrate PGx into routine workflows. Evaluating these tools in real-world settings should include clinical outcomes, such as reductions in adverse drug reactions, as well as cost-effectiveness analyses.

In addition, the development and evaluation of targeted training programs—particularly those incorporating real-world case scenarios—could help bridge knowledge gaps and enhance pharmacists’ readiness for implementation [43, 44]. Broader public awareness campaigns and the creation of harmonized regulatory frameworks encompassing reimbursement, data governance, and clinical guidance also warrant further exploration to support the wider adoption of PGx services [42, 45]

Future studies should also include pharmacists from settings where PGx has not yet been implemented, in order to identify context-specific barriers and inform inclusive implementation strategies. Moreover, conducting multi-country comparative studies using the questionnaire developed in this study—once formally validated—may offer valuable insights into how cultural and systemic differences affect PGx uptake. Finally, incorporating patient perspectives on PGx in the community pharmacy context is essential to ensure that implementation strategies are aligned with end-user needs and expectations.

## Conclusion

This study, based on the perspectives of LCs and KOLs, identified key barriers and facilitators to implementing PGx in pharmacy practice, with findings categorized post hoc using the CFIR framework. Structural barriers, such as fragmented ICT systems and a lack of reimbursement, continue to hinder progress, while professional motivation, education, and collaboration emerged as important facilitators. Embedding PGx into daily workflows, supported by practical tools and regional coordination, were considered essential facilitators for sustainable implementation. Although grounded in the Dutch context, these findings offer transferable insights for health systems aiming to integrate PGx into routine pharmacy care. Countries at earlier stages of PGx adoption may benefit from these lessons to anticipate structural challenges and proactively design supportive implementation strategies.

## Supplementary Information

Below is the link to the electronic supplementary material.

Supplementary file1 (DOCX 34 KB)

Supplementary file2 (DOCX 62 KB)

Supplementary file3 (DOCX 18 KB)

Supplementary file4 (DOCX 39 KB)

## Data Availability

The dataset generated and analyzed during the current study is available from the corresponding author upon reasonable request.

## Declarations

### Artificial intelligence

No generative Artificial Intelligence tools were used in the analysis, or interpretation of this manuscript. All text was produced by the authors themselves.
