# Supplementary material for: Barriers, facilitators, and implementation strategies for pharmacogenomics in community pharmacies: a cross-sectional survey among local champions in pharmacies and key opinion leaders in pharmacogenomics
Source: Int J Clin Pharm. 2025 Oct 23;48(2):544–56. doi: 10.1007/s11096-025-02022-x (PMC12992355; doi:10.1007/s11096-025-02022-x)
Supplement: Supplementary file 2 — Supplementary file2 (DOCX 62 KB) [file 11096_2025_2022_MOESM2_ESM.docx]

## Appendix I PGx questionnaire in pharmacy

## Introduction

Dear participant,

Welcome to our survey on pharmacogenetics in the pharmacy and the role of healthcare providers in this field. Your insights and experiences are invaluable in gaining a better understanding of current practices, challenges, and opportunities in pharmacogenetics within the pharmacy setting.

This survey aims to explore how you, as a healthcare provider, are involved with pharmacogenetics (PGx). Your responses will help us to develop a detailed picture of current activities, responsibilities within the pharmacy, methods of communication regarding medication safety, and the integration of PGx into patient care.

This questionnaire consists of a series of questions covering different aspects of PGx, including medication safety, documentation of reports, patient-centered approaches, communication, technological challenges, and collaboration with other healthcare providers. Your answers will serve as the foundation for the Invitational Conference on February 27, 2024.

We appreciate your time and expertise, and kindly ask that you provide additional explanations wherever possible. These clarifications are crucial for gaining deeper insights and will be used for further analysis and discussion.

The survey will take approximately 20 minutes to complete. Your responses will be treated anonymously and used only in preparation for the invitational and for research purposes.

Thank you in advance for your participation and valuable contributions to this research.

Kind regards,

Pantea Kiani, Pierre Bet, Petra Hoogland, Naomi Jesserun, Jeroen Mentink, Jesse Swen, and Sander Borgsteede.

Let’s begin!

## Personal Characteristics

1. What is your current position? (free text)
2. How many years have you worked in pharmacy?

- 0-5 years
- 6-15 years
- >15 years

1. Specialization: (free text)
2. Gender:

- Female
- Male
- Other: (free text)

1. Are you a practicing healthcare professional?

- Yes, in a community pharmacy

→*Questionnaire goes to ‘Role of Healthcare Professionals’*

- Yes, in a outpatient pharmacy

→*Questionnaire goes to ‘Role of Healthcare Professionals’*

- Yes, in a hospital pharmacy

→*Questionnaire goes to ‘Role of Healthcare Professionals’*

- Yes, in an institutional pharmacy

→*Questionnaire goes to ‘Role of Healthcare Professionals’*

- No, I work elsewhere: (free text)

→*Questionnaire goes to ‘Role of Non-Healthcare Professionals ’*

Role of Non-Healthcare Professionals

1. What is your role in the pharmaceutical sector? (free text)
2. Are you involved in decision-making regarding pharmacogenetics in your organization?

- Yes
- No

Comment: (free text)

1. How do you see the future impact of pharmacogenetics on healthcare?

- Negative
- Neutral
- Positive

Comment: (free text)

1. Do you see obstacles to the implementation of pharmacogenetics?

- Lack of evidence
- Costs
- Legal issues
- Other: (free text)

1. How do you evaluate current communication and collaboration between healthcare and non-healthcare professionals regarding PGx?

- Ineffective
- Neutral
- Very effective

Comment: (free text)

1. Are you aware of current PGx regulations in the pharmaceutical sector?

- No
- Partially
- Yes

Comment: (free text)

1. What is the role of non-healthcare professionals in promoting the use of PGx in healthcare?

- No important role
- Important role

Comment: (free text)

1. Have you been involved in PGx-related initiatives or policies?

- Yes
- No

Comment: (free text)

1. Do you have suggestions for improving the involvement of non-healthcare professionals in PGx initiatives?

- Yes
- No

Comment: (free text)

1. Do you think there is sufficient education and awareness about PGx among non-healthcare professionals?

- Yes
- No

Comment: (free text)

1. What is your vision of the future role of non-healthcare professionals in implementing PGx in the pharmaceutical sector?

- Active role
- Limited role
- No role

Comment: (free text)

1. Are there specific policies or initiatives you would like to see to strengthen this role?

- Yes
- No

Comment: (free text)

1. Are there any other topics or insights you would like to share that were not yet covered?

- Yes
- No

Comment: (free text)

## Role of Healthcare Professionals

1. Do you advice on medication safety in PGx?

- Yes
- No
- Other: (free text)

1. Approximately how many patients per week are affected by PGx in your practice? (number)
2. Who is responsible for advice on PGx-related medication safety in your pharmacy?

- Pharmacist
- Technician
- Both

Comment: (free text)

1. Are full PGx reports stored in the patient file and why?

- Yes
- No

Comment: (free text)

1. How is communication about advices on PGx and medication conducted?

- By local agreements
- Mainly case by case with GP or specialist
- Other: (free text)

1. Do you target specific patient groups? If so, which ones? (multiple answers possible)

- Cardiovascular
- Mental health
- Pain
- Oncology
- Other: (free text)

1. How often is dosage adjusted based on PGx?

- Never
- Rarely
- Regularly
- Always

Comment: (free text)

1. How is PGx information recorded in your pharmacy?

- Contraindication
- Lab result
- Both
- Other: (free text)

1. How do you receive PGx data from other healthcare professionals?

- Paper
- Fax
- Email
- Auto-registered as contraindication

Comment: (free text)

1. What other PGx services do you provide?

- PGx consultations
- Active patient outreach
- Participation in projects
- PGx is a subject in medication reviews
- Other: (free text)

1. From which laboratories do you receive PGx results? (free text)
2. What ICT challenges do you face in PGx implementation? (multiple answers possible)

- Manual work
- Difficulty identifying genotypes
- Data exchange issues
- Other: (free text)

1. How do you inform patients about PGx? (multiple answers possible)

- In-person
- Written materials
- Phone
- Website
- App

Comment: (free text)

1. Do you actively approach patients regarding PGx?

- Yes
- No

Comment: (free text)

1. What agreements have you made with other providers about PGx? (free text)
2. How have you promoted your visibility as a PGx expert?

- Posters in the pharmacy
- Journal articles in local media
- Other: (free tekst)

1. Does your pharmacy receive reimbursements for PGx-related services?

- Yes
- No

Comment: (free text)

1. What barriers do you experience in PGx implementation? (free text)
2. What facilitators do you experience? (free text)
3. What additional support do you need? (free text)
4. Are you aware of current guidelines and protocols on PGx?

- Yes
- Partially
- No

Comment: (free text)

1. How do you assess your PGx expertise level?

- Beginner
- Intermediate
- Expert

Comment: (free text)

1. Do patients seem well-informed about PGx? (free text)

- Yes
- Sometimes
- No

Comment: (free text)

1. How often do patients initiate consultations about PGx?

- Rarely/ never
- Sometimes
- Often
- Comment: (with possibly your estimate how often patients initiate a consultation)

1. How adequate are the current PGx regulations in your opinion?

- Inadequate
- Moderately
- Adequate

Comment: (free text)

1. Are there any specific PGx policies or initiatives you would like to see implemented?

- Yes
- No

Comment: (free text)

1. How do you see the future of PGx in your practice?

- Positive
- Uncertain
- Little developments
- Negative

Comment: (free text)

1. What PGx innovations or improvements would have the most impact on your practice?

- Technological improvements
- Better education and more knowledge among healthcare professionals
- More knowledge among medicine users
- More public awareness
- Better collaboration between healthcare professionals

Comment: (free text)

1. Are there other insights you wish to add? (free text)

## Closing

Thank you for completing this questionnaire. See you on the 27th!
