# Supplementary material for: Barriers, facilitators, and implementation strategies for pharmacogenomics in community pharmacies: a cross-sectional survey among local champions in pharmacies and key opinion leaders in pharmacogenomics
Source: Int J Clin Pharm. 2025 Oct 23;48(2):544–56. doi: 10.1007/s11096-025-02022-x (PMC12992355; doi:10.1007/s11096-025-02022-x)
Supplement: Supplementary file 3 — Supplementary file3 (DOCX 18 KB) [file 11096_2025_2022_MOESM3_ESM.docx]

Appendix III Mapping of PGx questionnaire

| **Domain** | **Questions** |
| --- | --- |
| Innovation, characteristics of the intervention | What is your vision on the position of PGx in healthcare?  How do you see the future of PGx?  What obstacles do you see for the implementation of PGx?  What initiatives or improvements will have the highest impact? |
| Inner setting | Do you store complete PGx-reports?  How is PGx information recorded?  What ICT problems do you experience concerning the implementation of PGx?  How is PGx information communicated with other ICT systems?  How many patients do you advise on PGx each week?  How do you inform your patients about PGx?  Who is responsible for advice on PGx?  Do you approach patients actively regarding PGx?  Do you advise on medication safety in patients with a PGx profile?  Do you aim your activities on a specific patient population? |
| Outer setting | Are you familiar with current regulations concerning PGx?  How adequate are current regulations for PGx, in your opinion?  Do you receive reimbursement for PGx-related services?  What is the importance of non-professionals in promoting PGx? |
| Individuals | How many patients do you advise on PGx each week?  How do you inform your patients about PGx?  Who is responsible for advice on PGx?  Do you approach patients actively regarding PGx?  Do you advise on medication safety in patients with a PGx profile?  Do you aim your activities on a specific patient population? |
| Implementation process | How do you assess the level of competence in pharmacogenetics?  How do you communicate with professional colleagues about PGx?  Are patients well-informed about PGx?  Is there sufficient awareness concerning PGx among non-healthcare professionals?  How do you rate current communication between healthcare professionals and non-professionals to PGx? |
