# Supplementary material for: Barriers, facilitators, and implementation strategies for pharmacogenomics in community pharmacies: a cross-sectional survey among local champions in pharmacies and key opinion leaders in pharmacogenomics
Source: Int J Clin Pharm. 2025 Oct 23;48(2):544–56. doi: 10.1007/s11096-025-02022-x (PMC12992355; doi:10.1007/s11096-025-02022-x)
Supplement: Supplementary file 4 — Supplementary file4 (DOCX 39 KB) [file 11096_2025_2022_MOESM4_ESM.docx]

# Appendix IV. Exemplary remarks and suggestions of respondents as illustrations of the comments and remarks

This appendix contains examples of remarks and suggestions made by respondents in response to one of the open-ended questions in the questionnaire.

In the Tables 2-5 in the manuscript, the most characteristic remarks/suggestions are presented. In this appendix, we illustrate these with a representative quotation. Any explanations of abbreviations are provided in square brackets []. Quotations were translated by the authors (SB and checked by XX) from Dutch to English.

| Table 2 Questions related to CFIR domain intervention characteristics. | | | |
| --- | --- | --- | --- |
| Question | Examples given in remarks and suggestions | Exemplary remark/suggestion by one of the respondents |  |
| What is your vision on the position of PGx in healthcare? | Improved quality, reduced costs, and quicker results anticipated | *Growing awareness and acceptance as added value.*  (Community pharmacist, local champion, more than 15 years of work experience, male) |  |
| How do you see the future of PGx? | Enhanced role of pharmacists, potential for cost reduction, and better patient outcomes | *More sustainable medication use, less trial and error, improved adherence, lower hospital costs.*  (Community pharmacist, local champion, 0–5 years of work experience, female) |  |
| What obstacles do you see for the implementation of PGx? | Barriers include funding issues, lack of ICT2 support, and insufficient knowledge. | *Inclusion in medical guidelines, data exchange, training (still needed)*  (Pharmacist/researcher, key opinion leader, work experience unknown, female) |  |
| What initiatives or improvements will have the highest impact? | Emphasis on improving technology, education, and regional cooperation | *National lobbying for reimbursement of consultations, testing, and national/regional knowledge networks.*  (Hospital pharmacist, local champion, 6–15 years of experience, female) |  |

| Table 3 Questions related to CFIR domains: outer setting and inner setting (organizational context) | | | |
| --- | --- | --- | --- |
| Question | Examples given in remarks and suggestions | Exemplary remark/suggestion by one of the respondents |  |
| Are you familiar with current regulations concerning PGx? | Need consistent frameworks | *Costs, laws and regulations, ICT [Information and Communication Technology] applications*  (Pharmacist, key opinion leader, 0–5 years of work experience, female) |  |
| Are you aware of current guidelines and protocols on PGx? | Need for clearer guidelines | *Not yet included in any guidelines to do this as standard*  (Community pharmacist, local champion, more than 15 years of work experience, male) |  |
| How adequate are current regulations for PGx, in your opinion? | Revision of existing regulations needed | *Privacy legislation is an additional obstacle to starting and conducting research. Government considers privacy much more important than the public does.*  (Hospital pharmacist, local champion, more than 15 years of work experience, gender: other) |  |
| Do you receive reimbursement for PGx-related services? | New reimbursement models suggested that overcoming financial barriers is critical. | *Reimbursement by health insurers is needed, and pharmacists should be able to request tests themselves and submit claims from the pharmacy.*  (Community pharmacist, local champion, more than 15 years of work experience, male) |  |
| What is the importance of non-professionals in promoting PGx? | Potential role in increasing public awareness and involvement | *Public campaigns.*  (Founding Director/pharmacist, key opinion leader, 6–15 years of work experience, male) |  |
| Do you store complete PGx-reports? | Need for consistent data storage | *Better sharing of results from hospitals to GPs[General Practitioners] /community pharmacies. Some patients have been genotyped, but I don’t know the result. Also record normal values in the PIS [Pharmacy Information System].*  (Community pharmacist, local champion, more than 15 years of work experience, male) |  |
| How is PGx information recorded? | Integration into existing systems recommended | *Electronic information directly in the PIS [Pharmacy Information System].*  (Community pharmacist, local champion, more than 15 years of work experience, female) |  |
| What ICT2 problems do you experience concerning the implementation of PGx? | High manual workload, difficulties in data exchange | *There is no mapping between the laboratory and the PIS [Pharmacy Information System], so everything has to be entered manually.*  (Community pharmacist, local champion, 6–15 years of work experience, male) |  |
| How is PGx information communicated with other ICT2 systems? | Electronic communication between systems needed | *Nationwide ICT [Information and Communication Technology] solutions are needed. Link tables. Data exchange between primary and secondary care must improve.*  (Community pharmacist, local champion, 6–15 years of work experience, male) |  |

| Table 4 Questions related to CFIR characteristics of the individuals | | | |
| --- | --- | --- | --- |
| Question | Examples given in remarks and suggestions | Exemplary remark/suggestion by one of the respondents |  |
| How many patients do you advise on PGx each week? | Encourage tracking and increasing patient advice based on PGx | *If you look at the number of people who have had pharmacogenetic testing and for whom action is required with prescribed medication: very limited. Not even every week. If you look at newly prescribed medication, for which you would actually want to test in advance: weekly; I’m thinking mainly of clopidogrel.*  (Community pharmacist, local champion, 6–15 years of work experience, female) |  |
| How do you inform your patients about PGx? | Enhance patient education efforts, especially in personalized settings. | *We often explain things to patients verbally and then provide written information for support.*  (Community pharmacist, local champion, >15 years of work experience, male) |  |
| Who is responsible for advice on PGx? | Establish clear roles within pharmacies. | *Flagging by pharmacy technicians, handling often by the pharmacist (depending on the alert’s handling level), and always checked afterward by the pharmacist.*  (Community pharmacist, local champion, >15 years of work experience, female) |  |
| Do you approach patients actively regarding PGx? | Strategies to actively engage patients | *Communication via app/website and leaflets.*  (Community pharmacist, local champion, >15 years of work experience, female) |  |
| Do you advise on medication safety in patients with a PGx profile? | Practical tools for integrating PGx into medication reviews are needed. | *Pharmacogenetics comes up during medication review.*  (Community pharmacist, local champion, 6–15 years of work experience, female) |  |
| Do you aim your activities on a specific patient population? | Patient-specific strategies for PGx use | *Depends on the indication. For research it is possible for multiple groups. Routinely: on request of the patient or prescriber in case of adverse effects. And often at the start of clopidogrel if not tested in hospital.*  (Pharmacist, local champion, >15 years of work experience, male) |  |

| Table 5 Questions related to CFIR domain process | | | |
| --- | --- | --- | --- |
| Question | Examples given in remarks and suggestions | Exemplary remark/suggestion by one of the respondents |  |
| How do you assess your PGx expertise level? | Comprehensive training programs required | *It is briefly covered in some training programs while it is complex subject matter. Additional training is also needed for pharmacists.*  (Pharmacist, key opinion leader, 6–15 years of work experience, female) |  |
| How do you communicate with professional colleagues about PGx? | Encouragement to establish local/regional hubs for better coordination | *Regionally, a guideline has been developed on how to request a pharmacogenetic test. FTO [pharmacotherapeutic consultation meetings] have been held to raise awareness among GPs [General Practitioners]. Pharmacists fill out the lab request form (on behalf of the GP and conduct the FG [PharmacoGenetics]-consultation.*  (Community pharmacist, local champion, 6–15 years of work experience, female) |  |
| Are patients well-informed about PGx? | Increase efforts to inform patients. | *It is getting more attention in the media. But we also explain it clearly to people ourselves.*  (Community pharmacist, local champion, >15 years of work experience, male) |  |
| Is there sufficient awareness concerning PGx among non-healthcare professionals? | Awareness campaigns needed | *It needs more attention in the media, on the radio.*  (Pharmacist, key opinion leader, 0–5 years of work experience, community pharmacy, female) |  |
| How do you rate current communication between healthcare professionals and non-professionals to PGx? | Standardized communication protocols needed | *Limitations of information systems and laboratories, no standardized coding system, limited national cooperation.*  (Pharmacist, key opinion leader, 6–15 years of work experience, female) |  |
